# A Glycosyltransferase-Related Signature for Predicting Overall Survival in Head and Neck Squamous Cell Carcinoma

**DOI:** 10.3389/fgene.2022.856671

**Published:** 2022-07-11

**Authors:** Huili Wu, Xiao Zhao, Tingting Zhu, Di Rong, Ying Wang, Diya Leng, Daming Wu

**Affiliations:** ^1^ Department of Endodontics, The Affiliated Stomatological Hospital of Nanjing Medical University, Nanjing, China; ^2^ Department of Oral and Maxillofacial Imaging, The Affiliated Stomatological Hospital of Nanjing Medical University, Nanjing, China; ^3^ Jiangsu Province Key Laboratory of Oral Diseases, Nanjing, China; ^4^ Jiangsu Province Engineering Research Center of Stomatological Translational Medicine, Nanjing, China

**Keywords:** glycosyltransferase, prognostic signature, head and neck squamous cell carcinoma, immune cell infiltration, tumor mutational burden

## Abstract

**Background:** Here, we establish a prognostic signature based on glycosyltransferase-related genes (GTRGs) for head and neck squamous cell carcinoma (HNSCC) patients.

**Methods:** The prognostic signature of GTRGs was constructed *via* univariate and multivariate Cox analyses after obtaining the expression patterns of GTRGs from the TCGA. A nomogram based on the signature and clinical parameters was established to predict the survival of each HNSCC patient. Potential mechanisms were explored through gene set enrichment analysis (GSEA) and immune cell infiltration, immune checkpoints, immunotherapy, and tumor mutational burden (TMB) analyses. The expression differences and prognostic efficacy of the signature were verified through the gene expression omnibus (GEO) and several online databases.

**Results:** The prognostic signature was constructed based on five glycosyltransferases (PYGL, ALG3, EXT2, FUT2, and KDELC1) and validated in the GSE65858 dataset. The pathways enriched in the high- and low-risk groups were significantly different. The high-risk group had higher tumor purity; lower infiltration of immune cells, such as CD8^+^ T cells and Tregs; higher cancer-associated fibroblast (CAF) infiltration; lower immune function; and lower checkpoint expression. The signature can also be applied to distinguish whether patients benefit from immunotherapy. In addition, the high-risk group had a higher TMB and more gene mutations, including those in TP53, CSMD1, CDKN2A, and MUC17.

**Conclusion:** We propose a prognostic signature based on glycosyltransferases for HNSCC patients that may provide potential targets and biomarkers for the precise treatment of HNSCC.

## Introduction

Head and neck squamous cell carcinoma (HNSCC) is the sixth most common neoplastic disease in humans, accounting for 90% of head and neck cancers (Wang et al., 2021). Every year, more than 830,000 people worldwide are diagnosed with head and neck cancers, most of which are advanced type, and 430,000 patients die from the disease. The 5-year survival rate of HNSCC patients is approximately 43% (Epstein et al., 2012; Chi et al., 2015). Approximately 40–60% of patients relapse even after a combination of surgery, radiation, and chemotherapy (Tolstonog and Simon, 2017). Therefore, it is important to evaluate the prognosis of HNSCC patients. The current evaluation for prognosis is mainly based on the TNM staging system, which includes assessing the primary tumor, lymph node metastasis, and distant metastasis and has limited accuracy (Chen et al., 2021). Therefore, it is necessary to construct a stable model to evaluate patient prognosis.

Glycosylation is a common posttranslational modification of proteins (Fournet et al., 2018). Several glycosidic linkages, including N-, O-, and C-linked glycosylation and glycophosphatidylinositol (GPI)-anchored attachment, are the main features of glycosylation (Rasheduzzaman et al., 2020). Glycosylation can modify the biological function of proteins, mainly affecting cell adhesion, migration, interactions with the cell matrix, cellular metabolism, cell signaling, and immune surveillance. Aberrant O-glycosylation was shown to be associated with tumor cell infiltration. For example, *α*-N-acetylgalactosamine (*α*-GalNAc) and *α*-2,6-sialyltransferase I (ST6GalNAc-I) overexpression could cause sialyl Tn (STn) expression disorder and C1GalT1-specific chaperone 1 (C1GALT1C1) mutation (Pinho and Reis, 2015).

The majority of current protein-based cancer biomarkers, such as PSA for prostate cancer or CA-125 for ovarian cancer, are glycoproteins (Almeida and Kolarich, 2016). Glycosylation was recognized to occur widely in tumor cells, resulting in the secretion of associated polysaccharides or glycoproteins, which serve as vital biomarkers, into the bloodstream (Silsirivanit, 2019). Glycosylation increases the heterogeneity and functional variability of tumor cells (Pinho and Reis, 2015), thus allowing tumor cells to have different glycan profiles at different stages of tumor growth and metastasis (Schjoldager et al., 2020).

At present, there are few studies on the construction of prognostic signatures based on glycosyltransferases. Therefore, we focused on glycosyltransferase to construct a signature in HNSCC patients using public databases to better distinguish their survival status. Furthermore, we explored pathway enrichment, immune cell infiltration, benefits of immunotherapy, and gene mutation status according to the signature.

## Materials and Methods

### Data Source

Transcriptome data, genomic mutation data, and corresponding clinical information from HNSCC patients, which contained 44 normal and 495 primary tumor tissues, were downloaded from The Cancer Genome Atlas (TCGA). GSE65858, containing 270 tumor samples with survival data, was obtained from the Gene Expression Omnibus (GEO) database to verify the prognostic signature. GSE30784 and GSE37991 were used to differentiate the expression levels of the genes in the signature between normal and HNSCC tissues. Moreover, we downloaded the expression data of one normal cell line (HaCaT) and six HNSCC cell lines (93VU147T, SCC61, SCC047, SCC090, SCC25, and SQ20B) from GSE62027. A total of 169 glycosyltransferase-related genes (GTRGs) were derived from a previous study (Mohamed Abd-El-Halim et al., 2021).

### Identification of Differentially Expressed GTRGs (DE-GTRGs) in HNSCC

A volcano plot and heatmap were used to visualize the DE-GTRGs, which were defined as those with *p* < 0.05 and |log2 (foldchange)| > 1. Then, a protein–protein interaction (PPI) network of DE-GTRGs was constructed by the GeneMANIA database to identify the interactions of glycosyltransferases. Moreover, the correlation between the expression of various DE-GTRGs was also analyzed.

### Construction of the Prognostic Signature

Univariate and multivariate Cox regression analyses were used to screen GTRGs to construct the prognostic signature. The signature reflects both the expression levels of the selected genes and their relative regression coefficient weights calculated from the multivariate Cox analysis. Patients in the training set were classified into high-risk and low-risk groups based on the median risk score. The Kaplan–Meier (KM) method was used to describe the differences in overall survival (OS) between the two groups. Receiver operating characteristic (ROC) curve analysis was further used to demonstrate the specificity and sensitivity of the signature. Principal component analysis (PCA) and T-distributed stochastic neighbor embedding (tSNE) methods were used to conduct dimension reduction analysis for all patients to evaluate the effect of the signature on the ability to distinguish between them. Finally, we incorporated the risk score and clinical parameters into the univariate and multivariate Cox regression analyses to prove that the risk score was an independent risk factor for prognosis.

### Correlation of Clinical Parameters and Risk Score

Clinical parameters including age, sex, tumor grade, tumor stage, T stage, and lymph node metastasis were used to perform a stratified analysis of OS. In addition, we analyzed differences in the risk score between subgroups based on the aforementioned clinical parameters.

### Nomogram and Calibration Curve Construction

A nomogram was established based on clinical parameters and the risk score. The calibration curves were drawn to assess the consistency between the actual and predicted probabilities of 1-year, 2-year, and 3-year survival. Furthermore, nomograms and calibration curves were also constructed according to the GSE65858 dataset.

### Gene Set Enrichment Analysis

GSEA was performed to identify the pathways enriched in the high-risk group or in the low-risk group according to the reference gene set Hallmark and KEGG analysis. An |NSE| > 1, a *p* value <0.05 and a false discovery rate (FDR) < 0.25 were set as the cut-off values.

### Immune Cell Infiltration, Checkpoints, and Immunotherapy Analyses Based on the Signature

The R package “ESTIMATE” was used to analyze the immune, stromal, and estimate scores and tumor purity between the high- and low-risk groups. TIMER, CIBERSORT, QUANTISEQ, MCPCOUNTER, and EPIC were used to compare the differences in immune cells between the two groups using the Wilcoxon test. Moreover, we analyzed the differences in immune cells and immune function between the two groups by using the ssGSEA method. Immune checkpoints, obtained from the TISIDB, were used to evaluate the differences between the two groups. Finally, to assess the value of the signature in patients receiving immunotherapy, multiple datasets were obtained to evaluate whether the signature could be used in immunotherapy patient cohorts. The cohorts included Van Allen’s cohort (CTLA-4 blockade in 40 metastatic melanoma patients) (Van Allen et al., 2015), Braun DA’s cohort (PD1 blockade in 311 advanced clear cell renal cell carcinoma patients) (Braun et al., 2020), Riaz N’s cohort (nivolumab in 50 advanced melanoma patients) (Riaz et al., 2017) and David Liu’s cohort (PD1 blockade in 121 metastatic melanoma patients) (Liu et al., 2019a).

### Tumor Mutational Burden and Gene Mutation Analysis

TMB was compared between the high- and low-risk groups and was used to analyze its prognostic value in HNSCC patients through KM analysis. In addition, survival analysis was performed according to TMB and the risk score. Gene mutation frequency was also analyzed between the two groups based on the mutation data from the TCGA, and the top 30 mutated genes are shown on a waterfall plot. We also obtained genes with significant mutation differences between the two groups.

### Identification of the Expression Levels and Prognostic Value of the Selected Genes

Immunohistochemistry images of PYGL, ALG3, FUT2, and KDELC1 were obtained from the HPA database. UALCAN was utilized to compare the protein levels of PYGL, EXT2, FUT2, and KDELC1 between normal and tumor tissues. Finally, a KM plotter was used to analyze the prognostic value of the five genes in HNSCC patients.

## Results

### Screening and Analysis of DE-GTRGs

**FIGURE 1:**
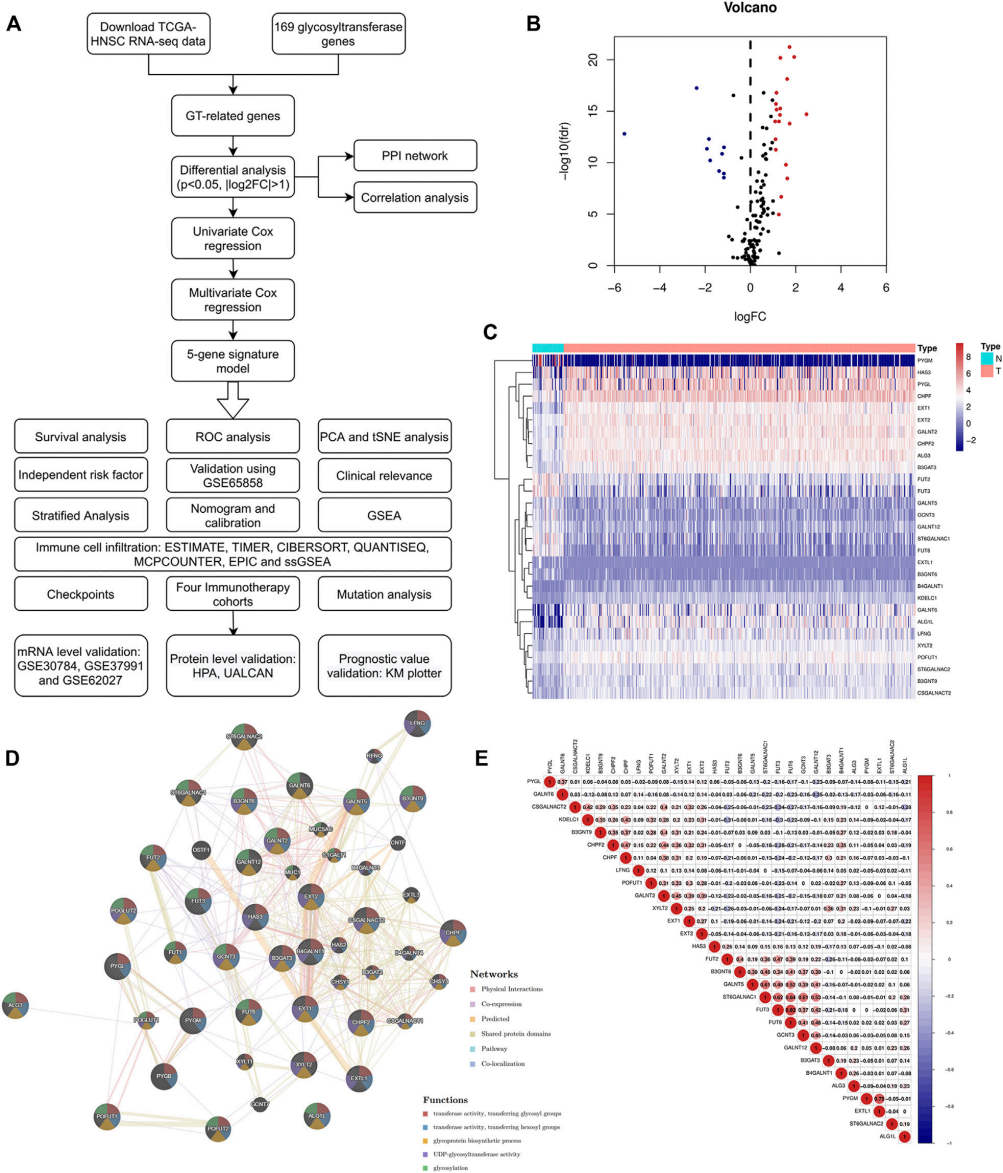
Identification of DE-GTRGs between 44 normal and 495 HNSCC tissues. **(A)** Brief flow chart of this study. **(B)** Volcano plot of 29 DE-GTRGs in TCGA-HNSCC. Red means upregulated and blue means downregulated genes. **(C)** Heatmap of DE-GTRGs between normal and HNSCC tissues. **(D)** PPI network based on the DE-GTRGs using the GeneMANIA database. **(E)** Correlation of the expression of DE-GTRGs in HNSCC tissues. DE-GTRGs, differentially-expressed glycosyltransferase-related genes; HNSCC, head and neck squamous cell carcinoma; TCGA, The Cancer Genome Atlas; PPI, protein–protein interaction.

A flow chart of the current study is shown in Figure 1A. We first obtained the expression values of 169 glycosyltransferase genes in normal and tumor tissues from the TCGA, and differential analysis was performed according to the abovementioned criteria. Twenty-nine DE-GTRGs were identified, among which 10 were downregulated and 19 were upregulated (Figures 1B,C). Based on the DE-GTRGs, a PPI network was constructed through the GeneMANIA database, and significant interactions were observed among these glycosyltransferases (Figure 1D). In addition, a generally positive or negative correlation was found among these DE-GTRGs (Figure 1E).

### Construction of a Prognostic Signature

**FIGURE 2:**
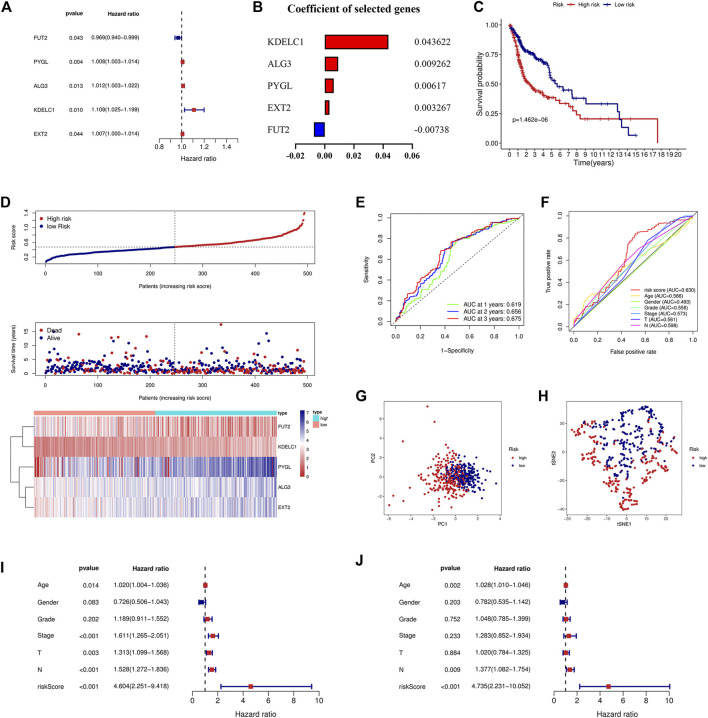
Construction of the prognostic signature based on five GTRGs. **(A)** Univariate Cox regression analysis identified five GTRGs associated with the prognosis. Red means risk genes and blue means protective genes. **(B)** Coefficients of the selected five genes in the signature through multivariate Cox analysis. **(C)** High-risk group had a worse prognosis than the low-risk group through the KM curve and log-rank test. **(D)** Risk score, survival time, survival status, and the relative expression of five genes between the high- and low-risk groups. **(E)** ROC curve analysis of the signature at 1-year, 2-year, and 3-year survival in the training cohort. **(F)** ROC curve analysis of the signature and the other clinical parameters in the training cohort. **(G)** PCA in the training cohort. **(H)** tSNE analysis in the training cohort. **(I)** Univariate Cox analysis of the signature and the other clinical parameters. **(J)** Identification of risk score as an independent risk factor for HNSCC patients through multivariate Cox analysis in the training cohort. KM, Kaplan–Meier; ROC, receiver operating characteristic; PCA, principal component analysis; tSNE, t-distributed stochastic neighbor embedding.

Univariate and multivariate Cox regression analyses were used to screen genes associated with patient prognoses and construct a prognostic signature. The results of the univariate analysis showed that five DE-GTRGs were associated with the prognosis of HNSCC patients, of which PYGL, ALG3, KDELC1, and EXT2 were risk factors and FUT2 was a protective factor (Figure 2A). Then, a prognostic signature based on five GTRGs was constructed utilizing multivariate Cox analysis, and the coefficients of the five genes are shown in Figure 2B. Risk score = (0.043622*KDELC1) + (0.009262*ALG3) + (0.00617*PYGL) + (0.003267*EXT2) - (0.00738*FUT2). The median risk score was applied to divide the HNSCC patients into a high-risk group and a low-risk group. Patients in the low-risk group had a better prognosis than those in the high-risk group (Figure 2C). As the risk score increased, there were more deaths and shorter survival times (Figure 2D). The areas under the curve (AUCs) of the signature at 1, 2, and 3 years were 0.619, 0.656, and 0.675, respectively (Figure 2E). The AUC of the risk score was 0.630, which was higher than that of the other six clinical parameters (Figure 2F). PCA and tSNE analyses were used to reduce dimensionality in all patients, and we found that patients with different risk scores could be distinguished significantly (Figures 2G,H). To analyze the prognostic value of the signature, univariate and multivariate analyses were used, and the results showed that the risk score was an independent risk factor for prognosis (Figures 2I,J).

### Validation of the Signature in an External Dataset

**FIGURE 3:**
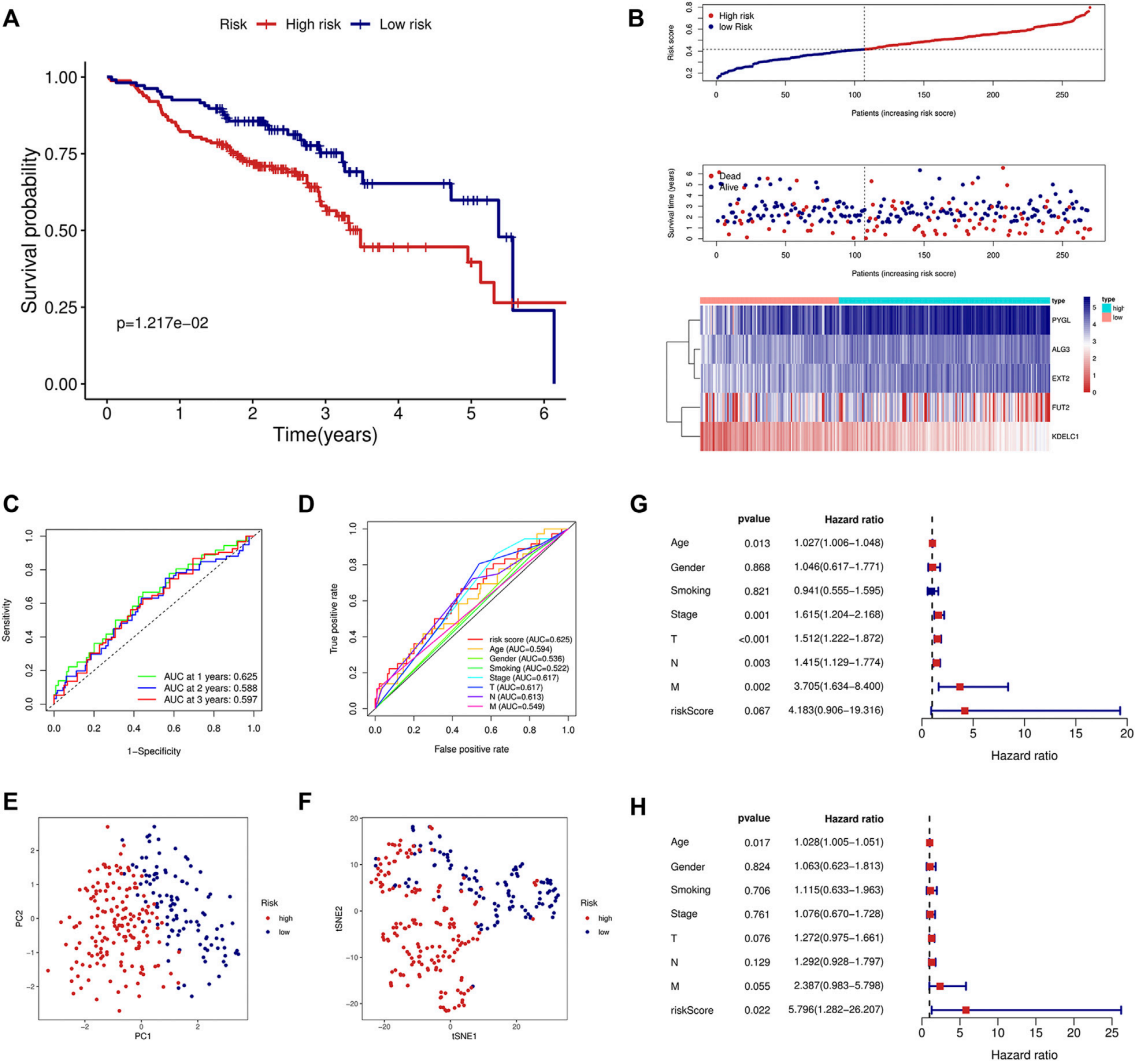
Validation of the prognostic signature in the GSE65858 validation cohort. **(A)** KM curve showed that the high-risk group had a shorter survival time than the low-risk group. **(B)** Distribution of the risk score, survival time, and survival status as well as the heatmap between the two groups in the validation cohort. **(C)** ROC analysis of the signature at 1-year, 2-year, and 3-year survival in the validation cohort. **(D)** ROC analysis of the signature and the other clinical parameters in the validation cohort. **(E)** PCA in the external validation cohort. **(F)** tSNE analysis in the external validation cohort. Univariate **(G)** and multivariate **(H)** Cox analyses of the signature and the clinical parameters in the validation cohort.

To validate the signature constructed through the TCGA, we obtained the expression profile and clinical parameters of the GSE65858 dataset. Similar to the training model, KM analysis showed poor prognosis in the high-risk group (Figure 3A). An increasing number of deaths were observed as the risk score increased (Figure 3B). ROC curve analysis revealed that the signature was of great value for patient prognosis. The AUCs of the signature at 1, 2, and 3 years were high, and the AUC of the risk score was 0.625, similar to the training model and higher than the other clinical parameters (Figures 3C,D). The results of PCA and tSNE analyses suggested that the prognostic signature can effectively distinguish high- and low-risk patients (Figures 3E,F). Again, the signature was an independent risk factor for patients in the external dataset (Figures 3G,H).

### Application of the Prognostic Signature in Clinical Subgroups

**FIGURE 4:**
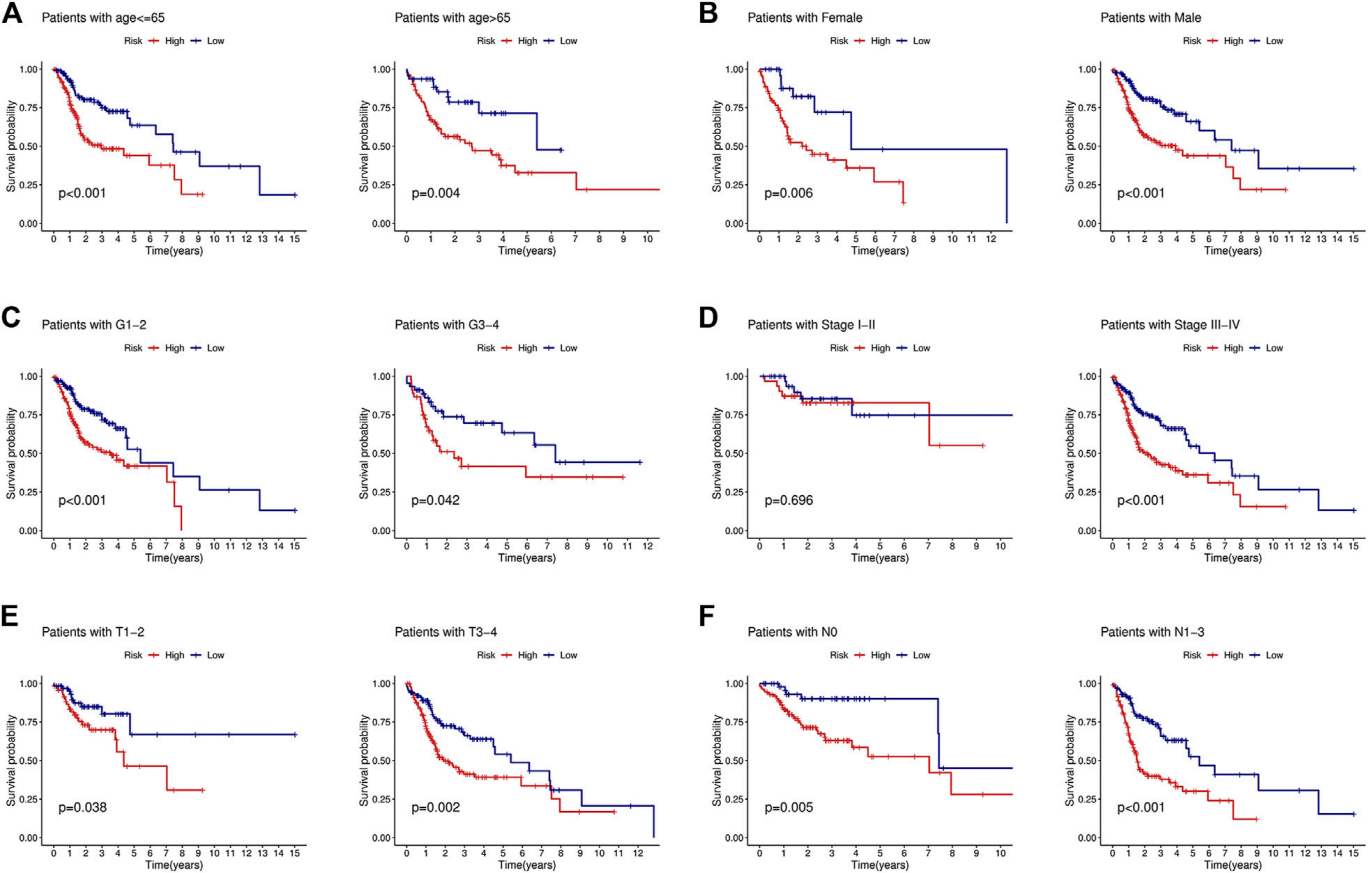
KM curves showed that the low-risk group had a better prognosis than the high-risk group stratified by the clinical features. **(A)** Age<=65 years and age>65 years. **(B)** Female and male. **(C)** Grades 1–2 and grades 3–4. **(D)** Stages I–II and stages III–IV. **(E)** T1-2 and T3-4. **(F)** N0 and N1-3.

To determine the value of the signature in different clinical subgroups, we performed a stratified analysis. We divided the patients into age ≤ 65 years, age >65 years, female, male, grades 1–2, grades 3–4, stages I–II, stages III–IV, T1–2, T3–4, N0, and N1–3 groups based on various clinical parameters (age, sex, tumor grade, tumor stage, T stage, and lymph node metastasis). The signature showed good efficacy in differentiating patient outcomes across all subgroups (Figure 4), indicating that the signature can be applied to all patients regardless of there being multiple clinical variables.

### Correlation With the Clinical Parameters and Construction of the Nomogram

**FIGURE 5:**
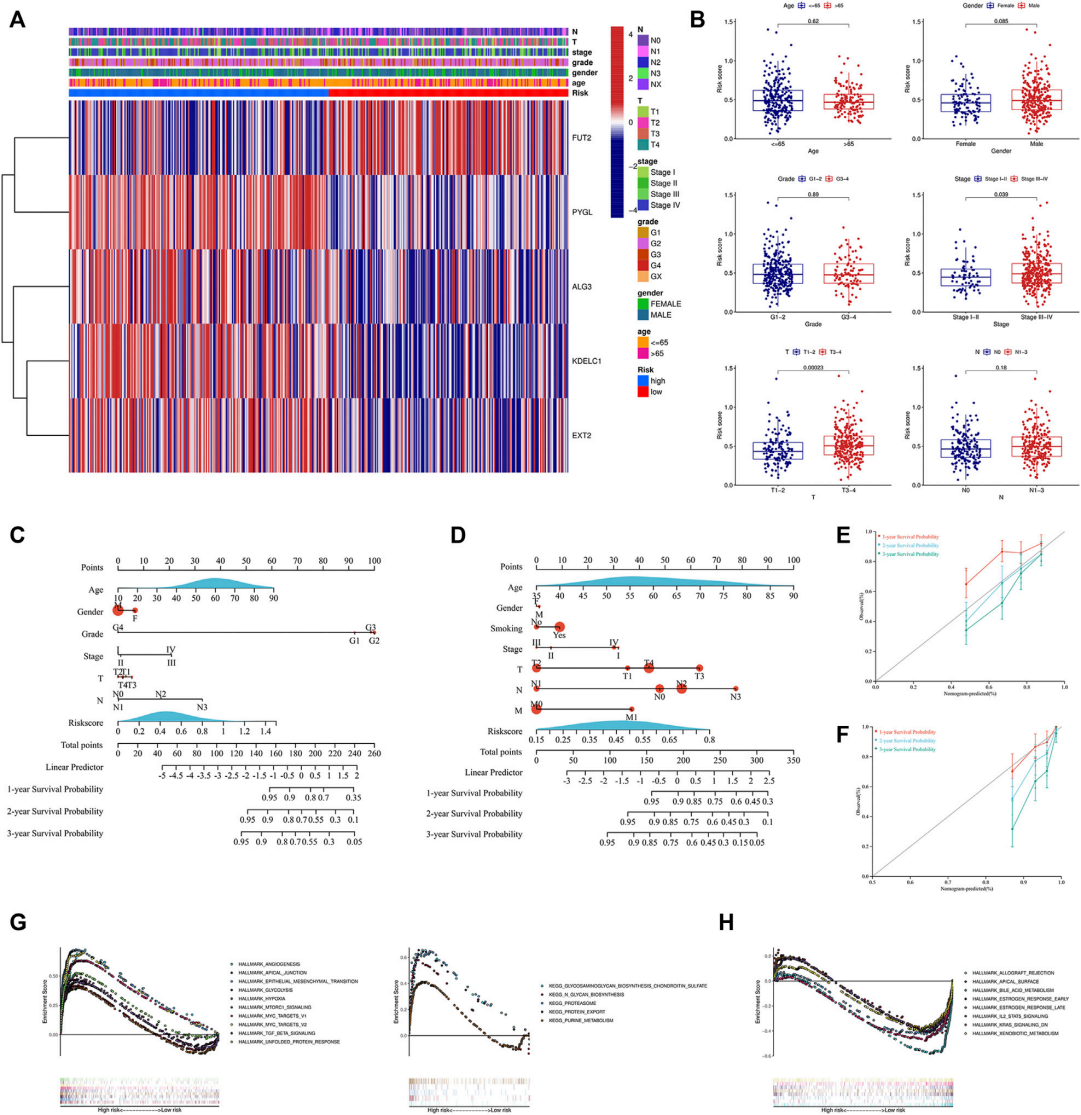
Analysis of the signature and clinical parameters. **(A)** Distribution of the clinical parameters and the expression of the five genes between the two groups. **(B)** Difference analysis of the risk score grouped by the clinical parameters. Construction of the nomogram combined with the risk score and the clinical parameters in the training cohort **(C)** and GSE65858 validation cohort **(D)**. Calibration curve of the nomogram at 1-year, 2-year and 3-year survival in the training cohort **(E)** and GSE65858 validation cohort **(F)**. Pathways enriched in the high-risk group **(G)** and low-risk group **(H)** through GSEA. GSEA, gene set enrichment analysis.

Due to the important value of the signature in different clinical subgroups, we then analyzed its correlation with clinical parameters. We found that the signature correlated significantly with tumor stage and tumor size; that is, in advanced tumors (stages III–IV) or when the tumor size was large (T3-4), the risk score was higher (Figures 5A,B). We also integrated the signature and clinical features to construct a nomogram. The nomogram was constructed to predict the 1-year, 2-year, and 3-year survival probabilities in the TCGA cohort (Figure 5C) and in the GSE65858 cohort (Figure 5D). The calibration curve showed good agreement between the predicted and actual 1-year, 2-year, and 3-year survival rates in both the training and validation cohorts (Figures 5E,F). GSEA showed that the high-risk group was associated with a variety of pathways related to tumor development, such as angiogenesis, epithelial–mesenchymal transition (EMT), glycolysis, hypoxia, and the TGF beta signaling pathway (Figure 5G), while the low-risk group was mainly enriched in IL2-STAT5 signaling, KRAS signaling, metabolism-related pathways, and others (Figure 5H).

### Differences in Immune Cell Infiltration and Immune Checkpoints

**FIGURE 6:**
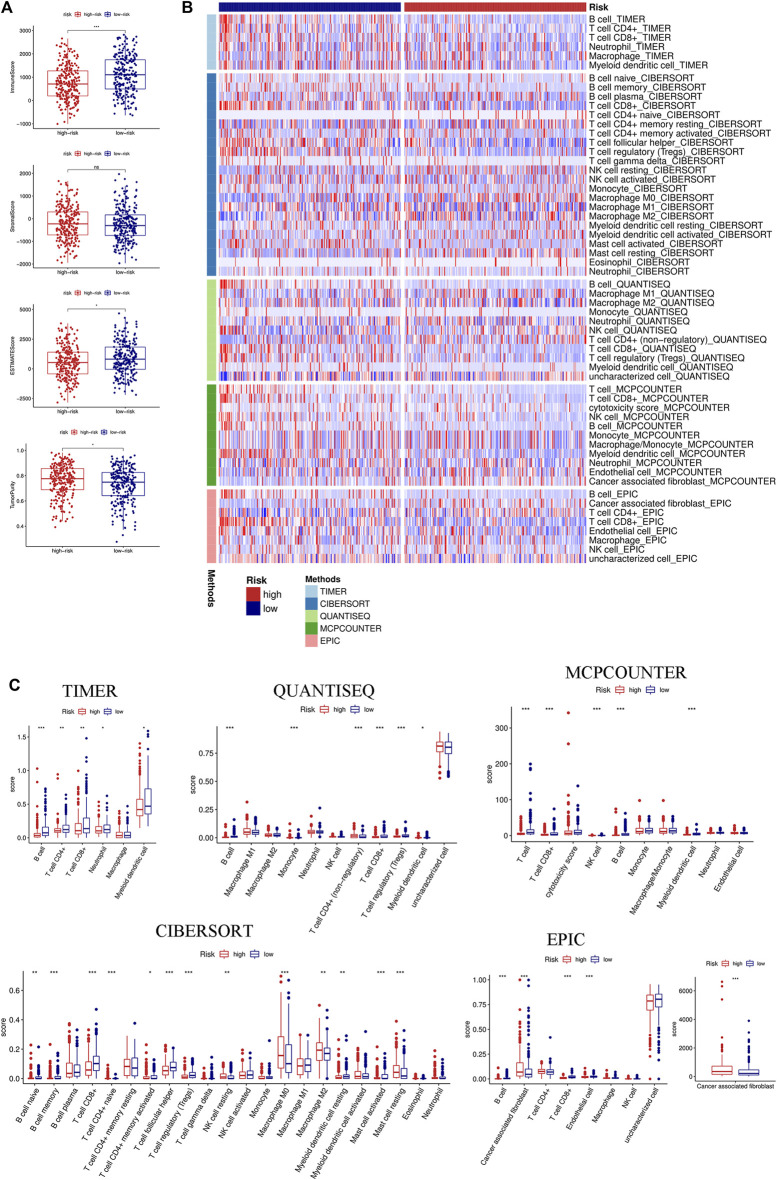
Immune landscape between the high- and low-risk groups based on multiple algorithms. **(A)** Comparison of the immune score, stromal score, ESTIMATE score, and tumor purity between the high- and low-risk groups based on the ESTIMATE algorithm. **(B)** Heatmap of the immune cell infiltration calculated by five algorithms, including TIMER, CIBERSORT, QUANTISEQ, MCPCOUNTER, and EPIC between the two groups. **(C)** Boxplot of the immune cell infiltration calculated by the abovementioned algorithms between the two groups. (*p < 0.05; **p < 0.01; ***p < 0.001; ns, not significant).

**FIGURE 7:**
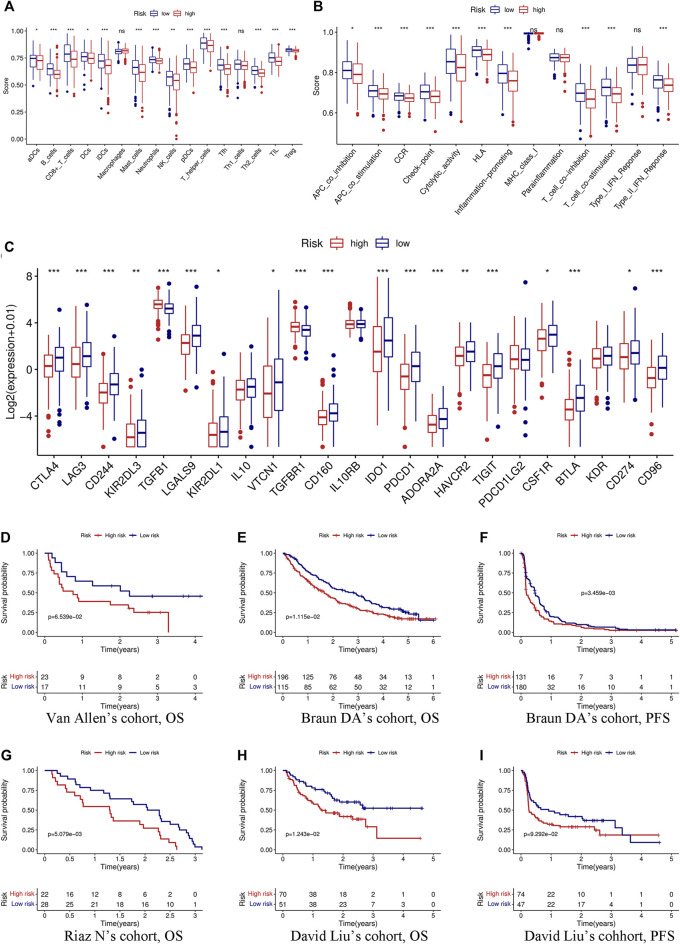
Immune function, immune checkpoints, and immunotherapy analysis based on the signature. Comparison of immune cells **(A)** and immune function **(B)** between the high- and low-risk groups through the ssGSEA algorithm. **(C)** Differences in the expression of 23 immune checkpoints between the high- and low-risk groups. **(D)** KM curve of OS in Van Allen’s cohort grouped by the signature. KM curves of OS **(E)** and PFS **(F)** in Braun DA’s cohort. **(G)** KM curve of OS in Riaz N’s cohort. KM curves of OS **(H)** and PFS **(I)** in David Liu’s cohort. OS, overall survival; PFS, progression-free survival. (*p < 0.05; **p < 0.01; ***p < 0.001; ns, not significant).

We first evaluated the immune score, stromal score, ESTIMATE score, and tumor purity. The immune and ESTIMATE scores were significantly lower in the high-risk group, while tumor purity was higher in the high-risk group (Figure 6A). We also compared the differences in immune cell infiltration between the two groups according to various algorithms. Based on the TIMER, CIBERSORT, QUANTISEQ, MCPCOUNTER, and EPIC, we found that B cells, CD8+T cells, dendritic cells, and Tregs were lower in the high-risk group and that cancer-associated fibroblasts (CAFs) were higher in the high-risk group (Figures 6B,C). In addition, we estimated the difference in immune cells and immune function between the two groups by using the ssGSEA algorithm. The results showed that immune cell infiltration was similar to that of the previous algorithm (Figure 7A), while multiple immune function scores were lower in the high-risk group, including checkpoints (Figure 7B). We, therefore, analyzed the differences between immune checkpoints in the two groups. Most checkpoints were significantly different between the two groups, and the expression levels of immune checkpoints were lower in the high-risk group, a pattern similar to that using the ssGSEA algorithm (Figure 7C).

### Immunotherapy Analysis According to the Signature

Immunotherapy has been considered to play an important role in a variety of malignant tumors and can markedly improve the prognosis. Therefore, it is necessary to identify people who may benefit from immunotherapy. We evaluated the value of the prognostic signature in immunotherapy for malignant tumors using four cohorts. Patients in the high-risk group had a shorter survival time (OS and progression-free survival) than those in the low-risk group in the four cohorts (Figures 7D–I).

### Relationship Between TMB and the Signature

**FIGURE 8:**
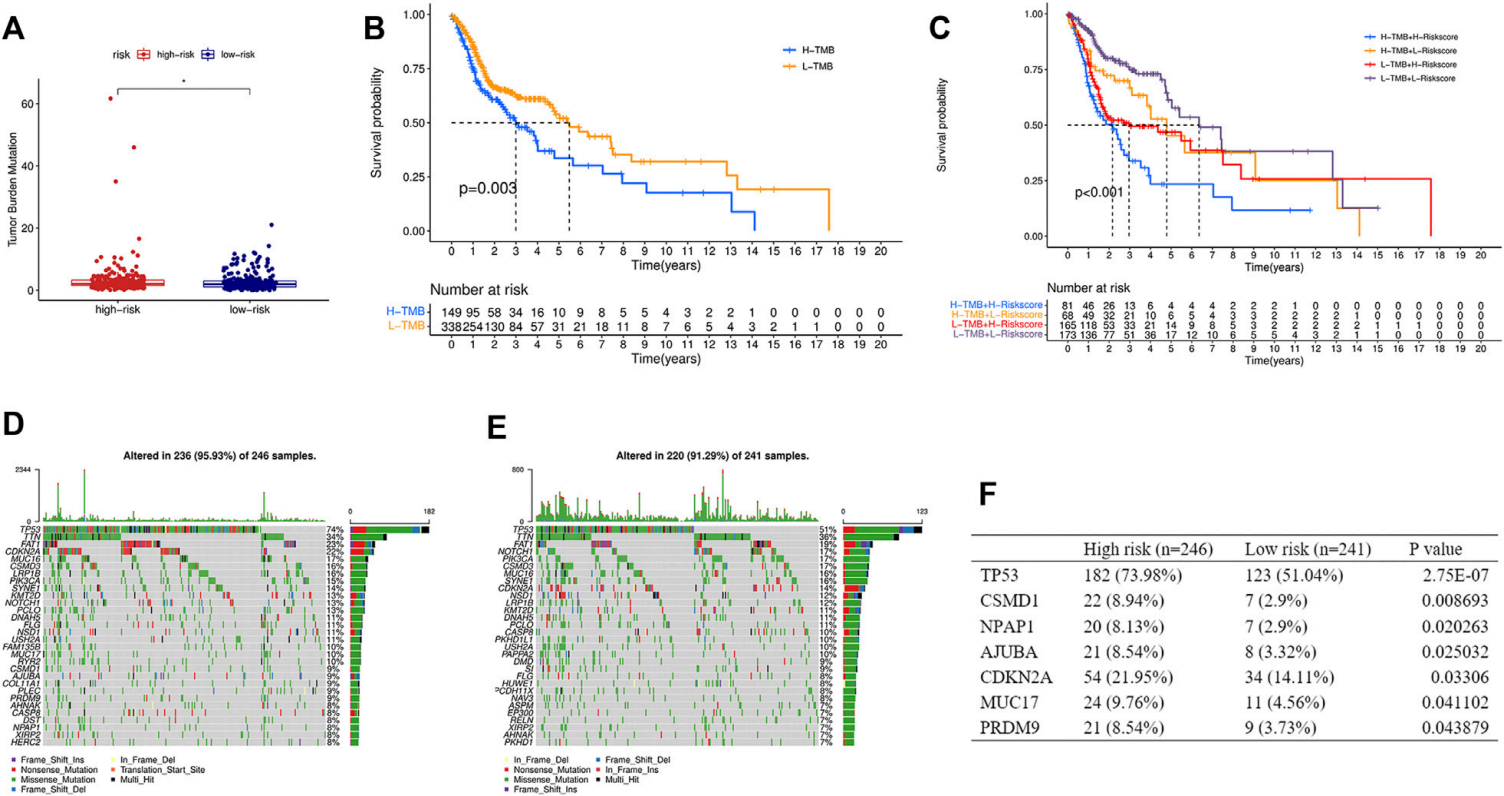
Relationship between the risk score and TMB. **(A)** Comparison of TMB between the high- and low-risk groups. **(B)** KM curve of the low-TMB and high-TMB groups. **(C)** KM curve of the HNSCC patients stratified by TMB groups and risk groups. Oncoplots displaying the top 30 somatic mutated genes in the high-risk group **(D)** and the low-risk group **(E)**. **(F)** Differences of the mutation frequency of the genes between the two groups. TMB, tumor mutational burden. (*p < 0.05).

TMB was calculated based on the somatic mutation obtained from the TCGA and was compared between the two groups. TMB in the high-risk group was higher than that in the low-risk group (Figure 8A), and the higher TMB group was associated with shorter survival time (Figure 8B). In addition, a stratified analysis based on TMB and the risk score showed significantly worse outcomes in the high-TMB + high-risk group than in the low-TMB + low-risk group (Figure 8C). Gene mutations visualized as a waterfall plot showed that the five most frequent somatic mutations in the high-risk group were those in TP53, TTN, FAT1, CDKN2A, and MUC16 (Figure 8D), while the five most frequent somatic mutations in the low-risk group were those in TP53, TTN, FAT1, NOTCH1, and PIK3CA (Figure 8E). Differential analysis between the two groups showed that the mutation frequencies of TP53, CSMD1, NPAP1, AJUBA, CDKN2A, MUC17, and PRDM9 were higher in the high-risk group (Figure 8F).

### Validation of the Selected Genes in Various Databases

**FIGURE 9:**
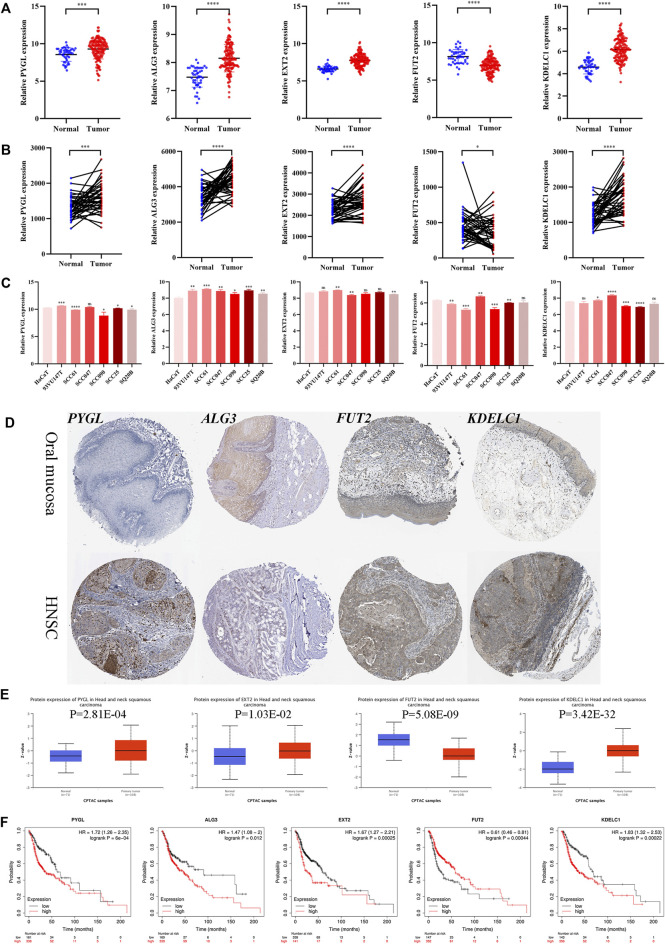
Expression and prognosis validation of the five glycosyltransferases. **(A)** Difference in the mRNA level of the five genes between 45 normal and 167 tumor tissues in the GSE30784 dataset. **(B)** Difference in mRNA level of the five genes between 40 matched normal and tumor tissues in the GSE37991 dataset. **(C)** mRNA level of the five genes in one normal cell line (HaCaT) and six HNSC cell lines (93VU147T, SCC61, SCC047, SCC090, SCC25, and SQ20B) in the GSE62027 dataset. **(D)** Immunohistochemistry images of the four glycosyltransferases in oral mucosa and HNSCC tissues from the HPA database. **(E)** Protein level of the four proteins between normal and tumor tissues in the UALCAN dataset. **(F)** KM curves of the five genes in HNSCC patients through the KM-plotter database. (*p < 0.05; **p < 0.01; ***p < 0.001; ****p < 0.0001).

The mRNA expression levels of the five genes in GSE30784 revealed that PYGL, ALG3, EXT2, and KDELC1 were upregulated while FUT2 was downregulated in HNSCC (Figure 9A). The differential expression analysis of the genes in the 40 paired normal and tumor tissues in GSE37991 showed similar results (Figure 9B). Furthermore, the expression level detected in one normal and six tumor cell lines was essentially consistent with that in tissues (Figure 9C). The immunohistochemistry images of PYGL, ALG3, FUT2, and KDELC1 in normal oral mucosa and HNSCC tissues are shown in Figure 9D. Meanwhile, the protein levels in the UALCAN database suggested that PYGL, EXT2, and KDELC1 were higher while FUT2 was lower in HNSCC tissues (Figure 9E). Finally, we performed survival analysis using KM curves in the KM plotter database. The results showed that the prognosis of patients with high expression of PYGL, ALG3, EXT2, and KDELC1 was worse, while high expression of FUT2 indicated longer survival time (Figure 9F).

## Discussion

Recently, due to advances in precision therapy in tumors, a variety of researchers have constructed prognostic models or identified molecular subtypes based on the associated gene sets of various malignant phenotypes. Qiu et al. constructed a prognostic model based on ferroptosis-related genes in pancreatic cancer and found possible correlations with different immune cells and classic immune checkpoints (Qiu et al., 2021). However, few researchers have constructed a prognostic model or identified subtypes based on GTRGs in different tumors. A previous study identified a series of glycosyltransferases and proposed a signature based on GTRGs to better classify pancreatic cancer patients with different prognoses and found the specific mechanisms of glycosylation in tumors and the microenvironment during tumor development (Mohamed Abd-El-Halim et al., 2021). Therefore, we aimed to identify the role of glycosyltransferases in HNSCC and construct a prognostic signature.

In this study, we identified a prognostic signature based on the five GTRGs (PYGL, ALG3, EXT2, FUT2, and KDELC1) through univariate and multivariate Cox regression analyses. The signature was proven to be an independent risk factor for HNSCC. In addition, the GSE65858 dataset was used to verify the prognostic performance of the signature, and we found that it can well-distinguish the prognoses of patients at high and low risk. We also found that the signature was closely related to immune cell infiltration and immune function. Patients in the high-risk group often had lower expression of immune checkpoints. Analyses of several immunotherapy cohorts also demonstrated that the signature can well-differentiate whether patients benefit from immunotherapy.

PYGL is a key phosphorylase that catalyzes the release of glucose molecules from glycogen (Han et al., 2018). A previous study indicated that an increased PYGL expression level was associated with increased tumor size in breast cancer, suggesting that PYGL may participate in tumor progression. *In vitro* and *in vivo*, hypoxia can induce the upregulation of glycogen metabolism and accumulation of glycogen in the early stage. The depletion of PYGL and decrease in glycogen accumulation decreased nucleotide synthesis and increased reactive oxygen species (ROS) levels, resulting in a decrease in breast cancer growth (Favaro et al., 2012). Several studies found that PYGL expression was upregulated in several cancers, including seminoma, brain cancer, and papillary renal cell carcinoma. KCNMB2-AS1 promotes esophageal cancer development by binding to miR-3194-3p and further upregulating PYGL expression (Xu et al., 2021). Numerous results have shown that PYGL is a vital target for anticancer therapy.

ALG3 is located on chromosomal region 3q27.1 and is a member of the mannosyltransferase family. Aberrant expression of several high-mannose type N-glycans during cancer progression has been increasingly identified (Munkley et al., 2016). Upregulation of ALG3 promoted the progression of cervical cancer (Choi et al., 2007) and non–small-cell lung cancer (Ke et al., 2020) and was proven to be associated with lymph node metastasis in esophageal squamous cell carcinoma (Shi et al., 2014). High ALG3 expression, negatively regulated by miR-98-5p, exerted a pro-carcinogenic effect by promoting EMT, thus leading to poor prognosis in non–small-cell lung cancer (Ke et al., 2020). In a 30-sample breast cancer cohort (including 15 radioresistant and 15 radiosensitive tumors), ALG3 was the most highly expressed of the ALG family in the radiation-resistant tissue. In addition, high ALG3 expression was associated with poor clinical parameters, short OS, and short relapse-free survival (Sun et al., 2021). A cancer stem cell–like (CSC) shape is thought to be the main cause of radioresistance (Knezevic et al., 2015). ALG3 can increase the radioresistance and tumor stemness of breast cancer cells and can upregulate several key CSC-like markers (Nanog, OCT4, and SOX2) by promoting the glycosylation of TGF-beta receptor II (Sun et al., 2021).

EXT2 is a member of the exotoxin glycosyltransferase family and is involved in the elongation of heparan sulfate (Ahn et al., 1995; Busse and Kusche-Gullberg, 2003). A large number of studies have found that mutations in EXT1 and EXT2 lead to loss of the protein domain, which is closely related to multiple osteochondromas (Guo et al., 2021; Tong et al., 2021). EXT2 was downregulated in breast cancer cells (Sembajwe et al., 2018) but upregulated in squamous cell lung carcinoma (Wu et al., 2021). Moreover, Huang et al. found that EXT2 was an independent risk factor for hepatocellular carcinoma (Huang et al., 2019).

Inactivating polymorphisms in FUT2, which encodes alpha 1,2-fucosyltransferase, were found to be associated with the increasing incidence of HNSCC (Campi et al., 2012; Su et al., 2016). FUT2 was decreased in HNSCC cells, and downregulation of FUT2 was related to a short survival time. EGFR was proven to be one of the potential alpha 1,2-fucosylated adhesion molecules (Montesino et al., 2021). In addition, FUT2 was upregulated and promoted cell migration and invasion in lung adenocarcinoma. A potential mechanism suggests that FUT2 may be involved in the TGF-beta/SMAD signaling pathway (Deng et al., 2018). The effect of FUT2 on tumor development and progression was also observed in breast cancer. Specifically, FUT2 can promote the proliferation, migration, and invasion of cells and is related to cell morphology changes, that is, from cuboidal to small and round cells (Lai et al., 2019). The expression of FUT2 was also downregulated by miR-15b and can facilitate the proliferation in hepatocellular carcinoma (Wu et al., 2014).

POGLUT2, formerly known as KDELC1 and homologous to POGLUT1, is a newly discovered protein O-glucosyltransferase that modifies sites different from POGLUT1 and can affect the Notch signaling pathway (Takeuchi et al., 2018). POGLUT2 was an independent prognostic factor and was used to construct a prognostic signature in clear cell renal cell carcinoma (Li et al., 2021), but few studies have examined the tumor mechanism.

Using multiple immune cell infiltration assessment algorithms, we found that the numbers of CD8^+^ T cells and Tregs were lower in the high-risk group, while the number of CAFs was higher. A previous study found that an increase in CD8^+^ T cells was an important prognostic indicator for OS in patients with relapsed HNSCC (So et al., 2020). Tregs play an important role in suppressing spontaneous tumor-associated antigen-specific immune responses (Oweida et al., 2019). Tregs were shown to be highly enriched in *in situ* HNSCC models and were associated with chemotherapy resistance (Oweida et al., 2018). Compared to healthy donors, HNSCC patients had increased tumor and blood Treg levels and lower CD8/Treg ratios. Indeed, high Treg and low CD8^+^ T-cell levels were considered poor prognostic factors for various tumors, including melanoma, ovarian cancer, colorectal cancer, and HNSCC. (Overacre-Delgoffe et al., 2017; Dolina et al., 2021), consistent with our results. CAFs are considered to be one of the most abundant mesenchymal cells and are observed in almost all types of solid tumors (Liu et al., 2019b; Chen and Song, 2019). Studies have shown that CAFs are associated with multiple biological oncogenic behaviors such as migration, invasion, self-renewal of tumor stem cells, chemotherapy resistance, and immune cell evasion (Zhang et al., 2013; Costa et al., 2018; Su et al., 2018). In oral squamous carcinoma, a higher density of CAFs suggests a more advanced tumor stage, a greater likelihood of lymph node metastasis, a greater incidence of local recurrence and distant metastasis, and a shorter survival time (Luksic et al., 2015). In addition, CAFs have been shown to play an important role in promoting HNSCC progression (Wheeler et al., 2014), mainly by secreting growth factors such as IL-6 and IL-8 (New et al., 2017), remodeling the extracellular matrix and enhancing therapeutic resistance (Bergers and Hanahan, 2008).

However, some deficiencies can also be found in our study. First, our data were based entirely on the public databases, such as the TCGA and GEO, and lacked experimental validation for the expression differences and prognostic model efficacy. Second, the pro- or antitumor phenotypes or mechanisms of the five genes have not been confirmed by *in vivo* or *in vitro* experiments. Third, the effect of the signature with respect to immunotherapy should be further examined using real-world data in future research.

## Conclusion

We proposed a prognostic signature for HNSCC patients constructed by incorporating five GTRGs from public databases. The high-risk group had lower immune CD8^+^ T cell and Treg infiltration but higher CAF infiltration. Furthermore, the signature can help judge prognostic differences in HNSCC patients and screen patients who may benefit from immunotherapy.

## Data Availability

The original contributions presented in the study are included in the article/Supplementary Material; further inquiries can be directed to the corresponding author.
